# Etiology of Severe Febrile Illness in Low- and Middle-Income Countries: A Systematic Review

**DOI:** 10.1371/journal.pone.0127962

**Published:** 2015-06-30

**Authors:** Namrata Prasad, David R. Murdoch, Hugh Reyburn, John A. Crump

**Affiliations:** 1 Centre for International Health, Dunedin School of Medicine, University of Otago, PO Box 56, Dunedin, 9054, New Zealand; 2 Department of Pathology, University of Otago Christchurch, 2 Riccarton Avenue, PO Box 4345, Christchurch, 8011, New Zealand; 3 Department of Disease Control, Faculty of Infectious and Tropical Diseases, London School of Hygiene and Tropical Medicine, Keppel Street, London, WC1E 7HT, United Kingdom; University of Texas Medical Branch, UNITED STATES

## Abstract

**Background:**

With apparent declines in malaria worldwide during the last decade and more widespread use of malaria rapid diagnostic tests, healthcare workers in low-resource areas face a growing proportion of febrile patients without malaria. We sought to describe current knowledge and identify information gaps of the etiology severe febrile illness in low-and middle-income countries.

**Methods and Findings:**

We conducted a systematic review of studies conducted in low-and-middle income countries 1980–2013 that prospectively assessed consecutive febrile patients admitted to hospital using rigorous laboratory-based case definitions. We found 45 eligible studies describing 54,578 patients; 9,771 (17.9%) had a positive result for ≥1 pathogen meeting diagnostic criteria. There were no eligible studies identified from Southern and Middle Africa, Eastern Asia, Oceania, Latin American and Caribbean regions, and the European region. The median (range) number of diagnostic tests meeting our confirmed laboratory case definitions was 2 (1 to 11) per study. Of diagnostic tests, 5,052 (10.3%) of 49,143 had confirmed bacterial or fungal bloodstream infection; 709 (3.8%) of 18,142 had bacterial zoonosis; 3,488 (28.5%) of 12,245 had malaria; and 1,804 (17.4%) of 10,389 had a viral infection.

**Conclusions:**

We demonstrate a wide range of pathogens associated with severe febrile illness and highlight the substantial information gaps regarding the geographic distribution and role of common pathogens. High quality severe febrile illness etiology research that is comprehensive with respect to pathogens and geographically representative is needed.

## Introduction

Fever is a common reason for seeking healthcare in low- and middle-income countries (LMICs) [1]. Among patients with febrile illness requiring admission case fatality ratios are high, sometimes exceeding 20% [2–6]. Fever etiology research [4,7,8] and the more widespread use of malaria diagnostic tests following changes to malaria treatment guidelines [9,10] have highlighted the problem of malaria over-diagnosis among patients with severe febrile illness. Apparent declines in malaria illnesses and deaths associated with malaria control efforts mean that the proportion of febrile patients with malaria has declined over the past decade [11,12].

While the global burden of disease due to diarrhea and pneumonia has been estimated at the syndrome level [13–15], such an approach has not been taken for fever without localizing features. Instead, illness and death due to some febrile diseases (e.g., dengue, malaria) are estimated [11,16], while others have been neglected (e.g., leptospirosis, Q fever). Comprehensive, standardized, and high quality, multi-center etiology research is being undertaken to understand the causes of severe childhood diarrhea and pneumonia [13,14] but such an approach has not been taken for fever. The many causes of fever are difficult to distinguish clinically [4,7,8] and laboratory services may be limited or absent in low-resource areas [17]. Consequently, clinicians frequently lack information about the local epidemiology of causes of severe febrile illness needed to adapt international management guidelines. Similarly disease control programs lack data to set priorities for prevention.

A robust contemporary picture of treatable and preventable infectious causes of severe febrile illness is urgently needed to improve patient outcomes and to inform disease control efforts in LMICs. Systematic reviews of studies of community-acquired bloodstream infections in Africa [18] and Asia [19] have demonstrated the importance invasive infections among febrile inpatients. A study mapping studies of the aetiology of non-malarial febrile illness in South East Asia [20] highlighted the diversity and geographical variation in a range of causes of fever. It also revealed the substantial information gaps that remain for a range of relevant pathogens.

To describe epidemiologic patterns and to identify data gaps in our understanding of severe febrile illness in low resource areas, we sought to systematically review prospective hospital-based studies of the etiology of febrile illness in LMICs.

## Methods

We followed Preferred Reporting Items for Systematic Reviews and Meta-Analyses (PRISMA) guidelines [21,22].

### Geographic and human development classification of countries

Countries were categorized into areas and regions according to the United Nations Population Division classification (Table 1) [23]. From each region, low- and middle-income countries were selected according to the 2012 Human Development index (HDI) [24].

**Table 1:** Etiology of severe febrile illness in low- and middle-income countries systematic review search terms.

Geographic terms			Etiology terms	
Area	Region	Country	Group	Pathogen (disease)
Africa	Eastern Africa	Burundi	Bacterial	(‘blood stream infections’/ ‘blood stream pathogens’/bacteremia/ bacteremia/septicemia/septicaemia fever/sepsis/’septic shock’)
		Comoros	Bacterial zoonoses	*‘Anaplasma phagocytophilum’/* (anaplasmosis)
		Djibouti		*‘Bartonella bacilliformis’/* (‘Carrión's disease’)/*’Bartonella henselae’/* (‘cat scratch disease’)/*’Bartonella Quintana’/* (‘trench fever’)
		Eritrea		*Borrelia /*(borreliosis)
		Ethiopia		*Brucella/*(brucellosis)
		Kenya		*Coxiella/* (‘acute Q fever’)
		Madagascar		*Ehrlichia*/(ehrlichiosis)
		Malawi		*Leptospira/* (leptospirosis)
		Mozambique		*‘Neorickettsia sennetsu’*
		Rwanda		*‘Orientia tsutsugamushi’*/(‘scrub typhus’)
		Somalia		*Rickettsia/* (murine typhus/’Spotted fever group rickett*/’Typhus group rickett*‘
		Seychelles		
		Tanzania/’United Republic of Tanzania’	Fungal	*‘Coccidioides immitis’/* (fungemia/mycoses/coccidiodomycosis)
		Uganda		*‘Cryptococcus neoformans’* (fungemia/mycoses/cryptococcosis)
		Zambia		*Histoplasma/* (fungemia/mycoses/ histoplasmosis)
		Zimbabwe		*Candida/*(fungemia/mycoses/ candidiasis/candidemia)
	Middle Africa	Angola		*‘Blastomyces dermatitidis’/* (fungemia/mycoses/blastomycoses)
		Cameroon		
		‘Central African Republic’	Viral	Dengue/(‘dengue fever’ ‘dengue hemorrhagic fever’/’DF’/’DHF’)
		Chad		‘Chikungunya virus’/ (chikungunya)
		Congo		‘Yellow fever virus’/ (‘yellow fever’)
		Congo/’Democratic Republic of the Congo’		‘West Nile virus’/ (‘West Nile’)
		‘Equatorial Guinea’		Influenza/(‘human influenza’)
		Gabon		‘Measles virus’/(measles)
		‘Sao Tome and Principe’		
	Northern Africa	Egypt	Blood parasite	*‘Plasmodium falciparum’/’Plasmodium malariae’/ ‘Plasmodium vivax’/* (malaria)
		Morocco		*‘Babesia microti’/*(babesiosis)
		‘South Sudan’		**‘** *Trypanosoma brucei rhodesiense’/’Trypanosoma brucei gambiense’ /’Trypanosoma cruzi’* /(‘African trypanosomiasis’)
		Sudan		‘*Leishmaniasis donovani’ /*(‘Visceral leishmaniasis’)
		Tunisia		
	Southern Africa	Botswana		
		Lesotho		
		Namibia		
		‘South Africa’		
		Swaziland		
	Western Africa	Benin		
		‘Burkina Faso’		
		‘Cape Verde’		
		‘Cote d'Ivoire’ /’Ivory Coast’		
		Gambia		
		Ghana		
		Guinea		
		‘Guinea-Bissau’		
		Liberia		
		Mali		
		Mauritania		
		Niger		
		Nigeria		
		Senegal		
		‘Sierra Leone’		
		Togo		
Latin America and the Caribbean	The Caribbean	‘Dominican Republic’		
		Haiti		
	Central America	Belize		
		El Salvador		
		Guatemala		
		Honduras		
		Nicaragua		
		Panama		
	South America	Bolivia		
		Guyana		
		Paraguay		
		Suriname		
Asia	South-Central Asia	Afghanistan		
		Bangladesh		
		Bhutan		
		India		
		Kyrgyzstan		
		Maldives		
		Nepal		
		Pakistan		
		Tajikistan		
		Turkmenistan		
		Uzbekistan		
	Eastern Asia	China		
		Mongolia		
	South-Eastern Asia	Cambodia		
		Indonesia		
		‘Lao People's Democratic Republic’		
		Myanmar/Burma		
		Philippines		
		Thailand		
		Timor-Leste		
		Viet Nam		
	Western Asia	Iraq		
		Jordan		
		‘State of Palestine’/ ‘Occupied Palestinian Territory’/ Palestine		
		‘Syrian Arab Republic’/ Syria		
		Yemen		
Oceania	Melanesia	Fiji		
		‘Papua New Guinea’		
		‘Solomon Islands’		
		Vanuatu		
	Micronesia	Kiribati		
		‘Micronesia’/ ‘Federated States of Micronesia’		
	Polynesia	Samoa		
		Tonga		
Europe	Eastern Europe			

* Truncated term used.

### Pathogens, diseases, and case definitions

Three investigators (NP, DRM, JAC) developed a list of pathogens and diseases associated with febrile illness in low- and middle-income countries (Table 1). Case definitions based on laboratory confirmation were used for each pathogen (Table 2).

**Table 2:** Case definitions for infections sought in systematic review of severe febrile illness in low- and middle-income countries.

Group	Disease	Confirmed case definition
Blood and tissue parasites	Babesiosis	Blood film and identification; serology with ≥4-fold rise in reciprocal titer between acute- and convalescent-phase serum specimens; NAAT
	Malaria	Blood film and identification; rapid diagnostic test; NAAT
	Trypanosomiasis	Blood film and identification
	Visceral leishmaniasis	Tissue biopsy or aspirate and identification
Invasive bacterial infections	Bloodstream infection	Blood culture and isolation; urine antigen testing for *Streptococcus pneumoniae* (adolescents and adults only) or *Legionella pneumophila* serogroup 1
Invasive fungal infections	Fungemia	Blood culture and isolation
	Blastomycosis	Fungal culture and isolation; antigen testing
	Candidosis	Fungal culture and isolation
	Coccidioidomycosis	Fungal culture and isolation; serology with ≥4-fold rise in reciprocal titer between acute- and convalescent-phase serum specimens; NAAT
	Cryptococcosis	Fungal culture and isolation; antigen testing
	Histoplasmosis	Fungal culture and isolation; antigen testing of urine or serum; ELISA; NAAT
Bacterial zoonoses	Anaplasmosis	Culture and isolation; serology with ≥4-fold rise in reciprocal titer between acute- and convalescent-phase serum specimens; NAAT
	Brucellosis	Culture and isolation; serology with ≥4-fold rise in MAT titer between acute- and convalescent-phase serum specimens
	Borreliosis	Culture and isolation; blood film; NAAT
	Cat scratch disease	Culture and isolation; serology with ≥4-fold rise in reciprocal titer between acute- and convalescent-phase serum specimens
	Carrión's disease	Culture and isolation; serology with ≥4-fold in reciprocal titer between acute- and convalescent-phase serum specimens
	Ehrlichiosis	Culture and isolation; serology with ≥4-fold in reciprocal titer between acute- and convalescent-phase serum specimens; NAAT
	Leptospirosis	Culture and isolation; serology with ≥4-fold in MAT titer between acute- and convalescent-phase serum specimens; NAAT
	Q fever	Culture and isolation; serology with ≥4-fold rise in IFA titer between acute- and convalescent-phase serum specimens; NAAT
	Scrub typhus	Culture and isolation; serology with ≥4-fold rise in IFA titer between acute- and convalescent-phase serum specimens, NAAT
	Spotted fever group rickettsiosis	Culture and isolation; serology with ≥4-fold rise in IFA titer between acute- and convalescent-phase serum specimens; NAAT
	Typhus group rickettsiosis	Culture and isolation; serology with four-fold or greater rise in IFA titer between acute- and convalescent-phase serum specimens; NAAT
	Trench fever	Culture and isolation; serology with ≥4-fold rise in reciprocal titer between acute- and convalescent-phase serum specimens
Viral infections	Dengue fever	Viral culture and isolation; NAAT; NS1; serology with ≥4-fold rise in reciprocal titer between acute- and convalescent-phase serum specimens;
	Chikungunya	Viral culture and isolation; NAAT; serology with ≥4-fold rise in reciprocal titer between acute- and convalescent-phase serum specimens
	Influenza	Viral culture and isolation; NAAT on nasopharyngeal and blood specimens; serology with ≥4-fold rise in HAI titer between acute- and convalescent-phase serum specimens
	Japanese B encephalitis	Viral culture and isolation; NAAT; serology with ≥4-fold rise in reciprocal titer between acute- and convalescent-phase serum specimens
	Measles	Viral culture and isolation; NAAT; serology with ≥4-fold rise in reciprocal titer between acute- and convalescent-phase serum specimens
	West Nile virus disease	Viral culture and isolation; NAAT; serology with ≥4-fold rise in reciprocal titer between acute- and convalescent-phase serum specimens
	Yellow fever	Viral culture and isolation; NAAT; serology with ≥4-fold rise in reciprocal titer between acute- and convalescent-phase serum specimens

NAAT = Nucleic acid amplification test; MAT = microagglutination test; IFA = immunofluorescence assay; HAI = haemagglutination inhibition assay.

### Search strategy and selection criteria

We searched three main databases: Ovid Medline, Scopus, and Web of Knowledge. The search included articles in all languages and was limited to articles investigating humans published from the year January 1980 through to July 2013. Search terms were identified and defined with the assistance of an academic liaison librarian (Sarah Gallagher) and are shown in Table 1. The search string combined the geography terms ‘country’ and etiology terms ‘pathogen’ or respective ‘disease’ (Table 1). For blood stream infections and rickettsial infections, only disease terms were searched without pathogen terms. Adjustments to the search strategy were made in accordance with the requirements of each database. Online translation tools were used to evaluate non-English titles, abstracts, and full text articles.

### Title and abstract review

One investigator (NP) reviewed titles and abstracts of articles identified by the search strategy. Those that appeared to be prospective studies of consecutive febrile patients enrolled in the emergency department or inpatient service of hospitals in an LMIC during the time period 1980 through 2013 were selected for full-text review. We excluded those that appeared to be: review articles, editorials, behavioural studies, economic impact studies, animal studies, vaccine and drug trials, diagnostic evaluations’ case reports, studies of persons not living in countries of interest such as travellers, or studies of outbreaks or epidemics. References for full-text review were compiled in Endnote version X6 (Thomson Reuters, Philadelphia, PA, USA), after the removal of duplicates, all articles were sought locally and internationally.

### Full-text review

Two investigators (DRM, JAC) reviewed full-text articles identified by the title and abstract review. When required, the third investigator (NP) served as tiebreaker, independently reviewing articles to resolve disagreement between the other two investigators. To be eligible for data extraction, full-text articles were confirmed to be prospective studies of consecutive febrile patients enrolled in the emergency department or inpatient service of hospitals in a low- or middle-income country during the time period 1980 through 2013. For the purposes of this review, febrile patients were defined as a person with a history of fever in the past 48 hours; an axillary temperature ≥37.5°C; or a rectal temperature ≥38.0°C. In addition, participants in such studies needed to be evaluated for at least one of the febrile diseases of interest using laboratory-confirmed case definitions (Table 2). We excluded studies of syndromes other than fever; studies of specific subgroups of febrile patients, such as HIV-infected persons; studies of health-care associated infections or studies where such infections could not be distinguished; and studies of outpatients or where outpatients and inpatients could not be distinguished.

### Validity assessment

We ensured the validity of the review by adhering to the predefined selection criteria to allow comparison across individual studies. By creating pre-determined case definitions we sought to capture only confirmed cases of infection. However, some variation in microbiological techniques and interpretation of results was unavoidable. We did not exclude studies on the basis of incomplete description of laboratory techniques, blood culture contaminants isolated, or failure to report all pathogens that may have been isolated or identified.

### Data extraction

The following data were extracted from each eligible study by one investigator (NP): geographical location of the healthcare facility; healthcare facility rurality; study time dates and duration; study inclusion and exclusion criteria including age range; diagnostic techniques for each infection; number of patients tested for each infection; number tested meeting case definition for each infection; use of additional tests (e.g., HIV serology). When available, we also recorded clinical diagnosis of patients; in-hospital fatality ratio; seasonal variation of pathogens; and pre-admission use of antimicrobials. For the purpose of this review, pediatric studies were defined as those that included patients aged from ≥28 days to <15 years. Studies with mixed populations of adults and children were analyzed as adult studies. Queries regarding data extraction were resolved by return to the original manuscript by three investigators (NP, DRM, JAC).

### Statistical analysis

Following data extraction, infections were organized into four groups: blood parasites; bacterial and fungal bloodstream infections; bacterial zoonoses; and viral infections, as shown in Table 2. Data from all individuals in all studies were aggregated to compare prevalence of febrile diseases across studies and regions. Summary statistics were calculated for key variables. Analyses of associations between patient factors or clinical conditions (e.g., HIV infection) and specific febrile diseases were done for studies with data for both the pathogens and factors being assessed. Chi-squared test was used to establish significance of associations and values were expressed as odds ratios (ORs) calculated with STATA software version 13.0 (College Station, TX, USA).

### Role of the funding source

The funders had no role in study design, data collection and analysis, decision to publish, or preparation of the manuscript. The corresponding author had full access to all the data in the study and had final responsibility for the decision to submit for publication.

## Results

### Search results

**Fig 1:**
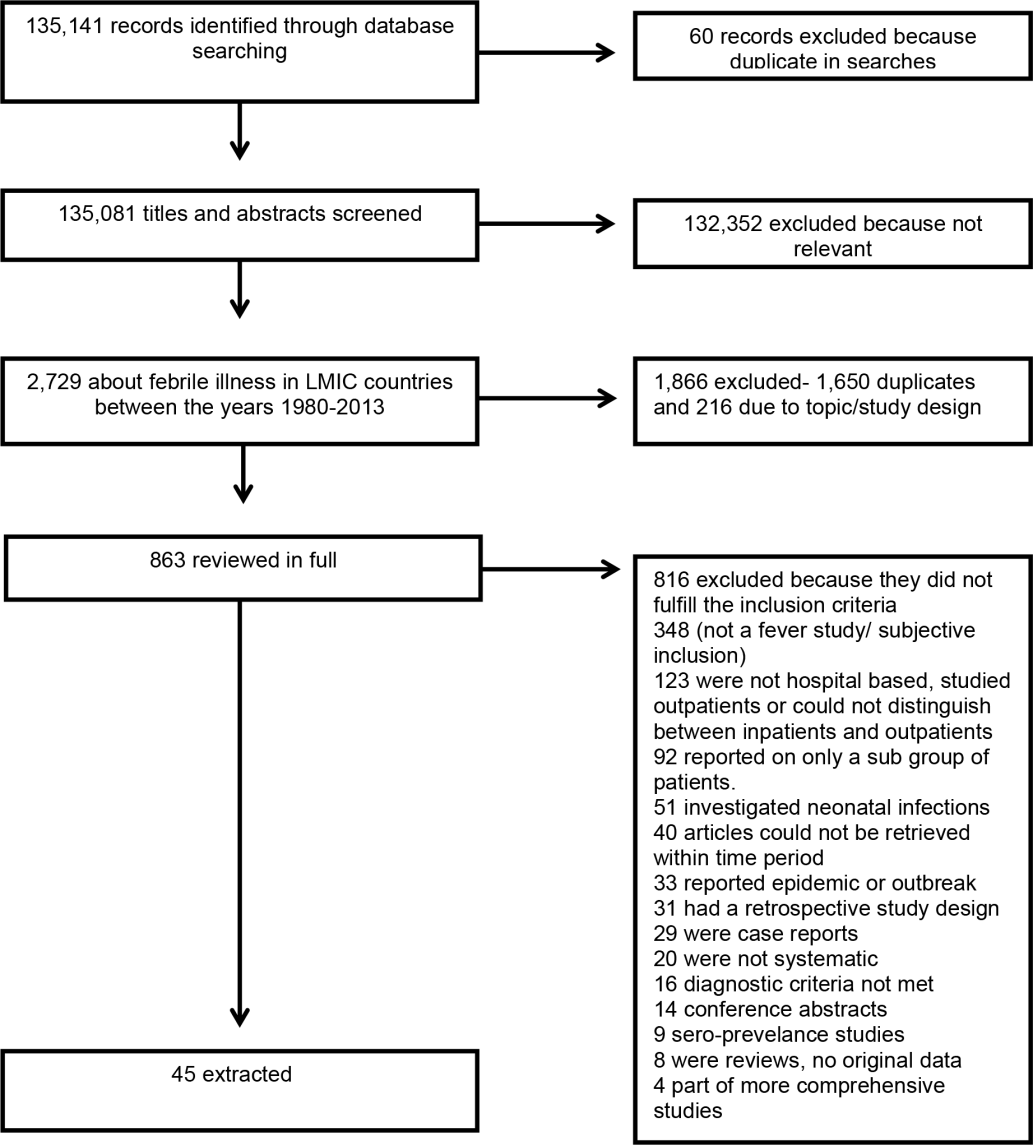
PRISMA flow diagram of selection of reports, systematic review of etiology of severe febrile illness in low- and middle-income countries, 1980–2013.

The online search completed on 28 September 2013 yielded 135,141 records of which 2,729 articles that appeared to be about febrile illness among humans in LMIC were selected. Of these 863 met criteria for full text review, 823 (95.4%) were available for full-text review of which, 45 (5.5%) were eligible (Fig 1).

### Characteristics of studies and patients

**Fig 2:**
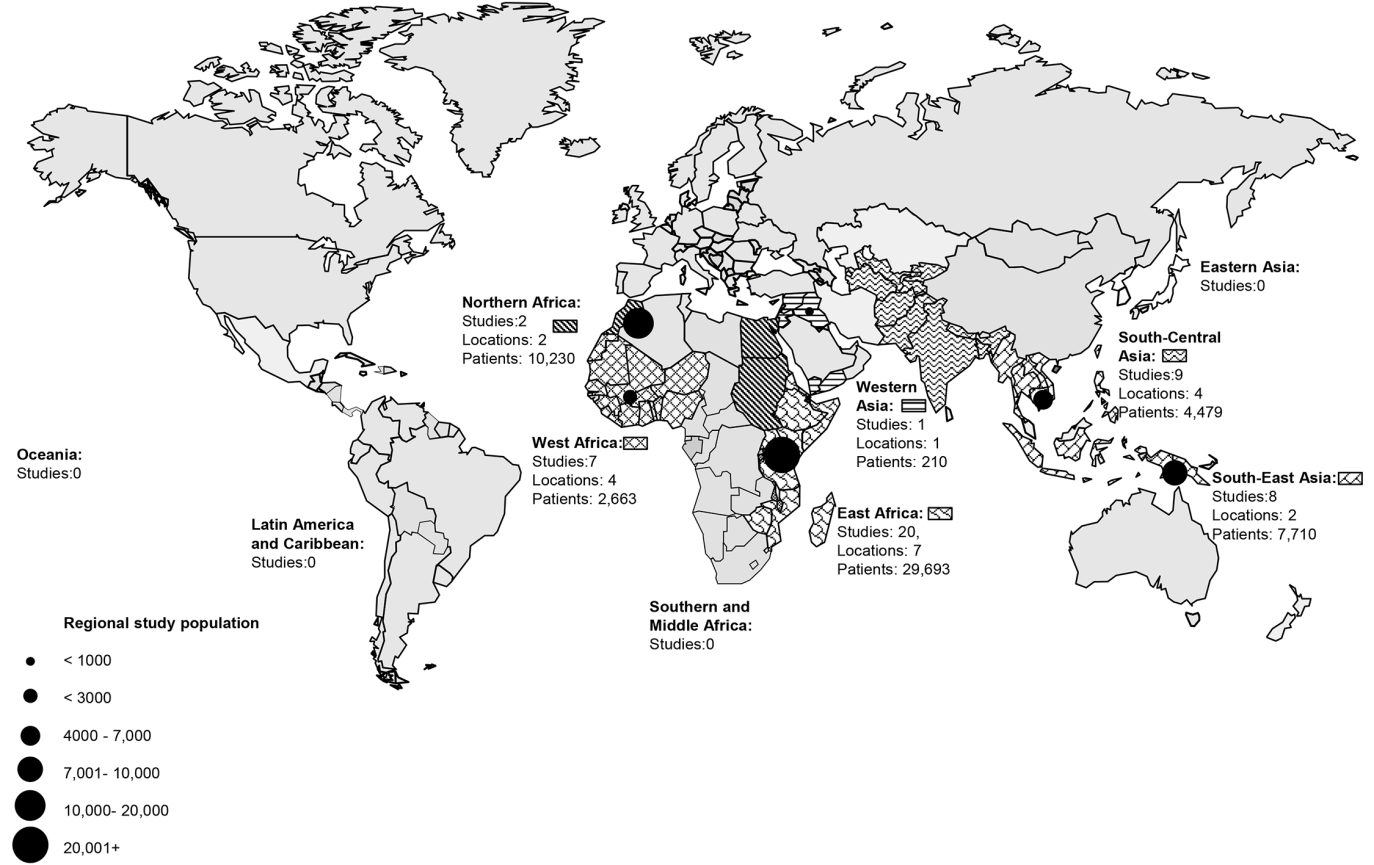
Febrile illness etiology study locations by United Nations population division regions in low- and middle-income countries, 1980–2013.

The 45 eligible studies were done in 22 locations and included 54,578 patients tested according to at least one laboratory-based case definition. Of all patients, 29,286 (53.7%) were from Eastern Africa; 10,230 (18.7%) from North Africa; 2,663 (4.9%) from Western Africa; 4,479 (8.2%) from South Central Asia; 7,710; (14.1%) from South East Asia; and 210 (0.4%) from Western Asia. There were no eligible studies identified from Southern and Middle Africa, Eastern Asia, Oceania, Latin American and Caribbean regions, and the European region (Fig 2).

### Infections searched meeting laboratory case definitions

Of the 25 febrile illnesses searched for in this review (Table 2), 16 (64.0%) were investigated according to our predetermined laboratory case definitions by at least one eligible study. Of studies, 22 (48.9%) investigated a single cause of febrile illness according to our selection criteria and laboratory case definitions.[5,6,25–43] The median (range) number of diagnostic tests meeting our confirmed laboratory case definitions was 2 (1 to 11) per study. Of the 45 eligible studies, 8 (17.8%) studies did not meet our inclusion criteria for all of the infectious diseases investigated in the study, with results for those diseases excluded from our analysis (Table 3) [3,25,29,30,36,44–46].

**Table 3:** Summary of 45 eligible studies of etiology of severe febrile illness in low- and middle-income countries, 1980–2013.

First Author (Reference)	Location; study dates	Total no. of patients in study	Hospital type	Age (population type)	Diagnostic tests conducted	N (%) of diseases searched in review investigated in study	Patients (%) with confirmed infection	Patients infected with HIV (proportion of patients tested)	Most common pathogens
**Eastern Africa**									
Aarsland, S. J. et al[44]	Ethiopia, December 2009—January 2010	102	Urban referral hospital.	1 month –18 years. Primarily children.	DNA extraction and NAAT from malaria blood smears for *S*. *pneumoniae*, *Salmonella* spp, *Rickettsia* spp, *Borrelia* spp, *Leptospira* spp. (NAAT for *Salmonella* and *S*.*pneumoniae* did not meet case definitions)	3 (12.0%)	12 (11.8%) with positive NAAT*		*Plasmodium* spp, *Rickettsia* spp, *Borrelia* spp*
Archibald, L. K., et al[2]	Tanzania; February 1995-April 1995	517	Urban referral hospital	>15 years.	Blood culture. Thick and thin blood smears	2 (8.0%)	145 (28.9%) positive blood culture. 49 (9.8%) malaria slide positive	282 (56.2%)	*Mycobacterium tuberculosis*, Non-typhoidal *Salmonella*, *S*.*aureus*,
Archibald, L. K., et al[56]	Malawi; July 1998—August 1998	229	Urban referral hospital.	1 month–13 years	Blood culture. Thick and thin blood smears	2 (8.0%)	35 (15.3%) positive blood culture. 13 (5.7%) malaria slide positive	63 (28%)	Non-typhoidal *Salmonella*, *E*.*coli*, *Acinetobacter*
Bell, M., et al[47]	Malawi; March 1998—May 1998	238	Urban referral hospital.	>14 years. Primarily adults	Blood culture (mycobacteria), Thick and thin blood smears	2 (8.0%)	67 (28.2%) positive blood culture. 72 (31.2%) malaria slide positive	173 (75.9%)	Non-typhoidal *Salmonella*, *Mycobacterium tuberculosis*, *Cryptococcus neoformans*
Christopher, A., et al[48]	Tanzania; September 2011—Feb 2012	317	Urban referral hospital.	2–60 months	Blood culture. Thick and thin blood smears	2 (8.0%)	21 (6.6%) positive blood culture. 82 (25.9%) malaria slide positive		*Plasmodium falciparum*, *E*.*coli*, *Klebsiella* spp.
Dougle, M., et al[51]	Kenya; July 1994—October 1994	228	Urban referral, teaching hospital.	> 5 years. Primarily adults	Blood culture. Thick and thin blood smears	2 (8.0%)	51 (22.4%) positive blood culture. 25 (11.0%) malaria slide positive	51 (22.5%)	*S enterica* serotype Typhi, *S*.*pneumoniae*, Non-typhoidal *Salmonella*
Gordon, M. A., et al. [5]	Malawi; December 1997—November 1998	9,298	Urban referral teaching hospital.	Unspecified. Primarily adults	Blood culture	1 (4.0%)	449 (16.1%) positive blood culture		Non-typhoidal *Salmonella*, *S*.*pneumoniae*, *E*.*coli*
McDonald, L. C., et al[35]	Malawi; August—September 1997	128	Urban referral hospital (Malawi)	> 18 years	Mycobacterial blood culture	1 (4.0%)	14 (10.9%) positive blood culture	101 (78.9%) in Malawi.	*Mycobacterium tuberculosis*
Meremo, A., et al[52]	Tanzania; June 2011—December 2011	346	Urban tertiary referral hospital.	Unspecified. Primarily adults	Blood culture	1 (4.0%)	33(9.5%) positive blood culture	156 (45.0%)	Non-typhoidal *Salmonella*, *S*.*pneumoniae*, *E*.*coli*
Nadjm, B., et al[53]	Tanzania; July 2006—May 2007	3,639	District, rural hospital	2 months—13 years.	Blood culture, malaria rapid diagnostic test, thick and thin blood smears	2 (8.0%)	341 (9.4%) positive blood culture. 2195 (60.3%) malaria slide positive	142 (3.9%)	Non-typhoidal *Salmonella*
Petit, P. L. C., et al[54]	Kenya, 1990	336	Study 1 urban and referral	> 8 years. Primarily adults	Blood culture, thick and thin blood smears	2 (8.0%)	Only study 1–104 (30.9%) positive BSI. 25 (7.4%) malaria slide positive	12 (3.6%)	*Plasmodium* spp, *Salmonella* spp, *E*.*coli*
Sigaúque, B., et al[40]	Mozambique; May 2001- April 2006	18,944	Rural district hospital	<15 years	Blood culture. Thick and thin blood smears (Blood smears included neonates)	2 (8.0%)	1395 (7.4%) true positive blood culture. 9939 (52.5%) with malaria slide positive		Non-typhoidal *Salmonella*, *S*.*pneumoniae*, *E*.*coli*,
Ssali, F. N., et al[6]	Uganda; January 2007—April 2007	299	Urban referral, hospital	>15 years.	Blood culture (mycobacterial)	1 (4.0%)	71 (23.7%) positive blood culture	228 (76.3%)	*Mycobacterium tuberculosis*, *S*.*pneumoniae*
Strøm, G. E. A[41]	Tanzania; January 2009- June 2009	304	Urban referral hospital.	1 month- 7 years.	Thick and thin blood smears, malaria rapid diagnostic test, NAAT	1 (4.0%)	76 (25.0%) NAAT positive for malaria		*Plasmodium falciparum*
Lofgren, S. M., et al[34]	Tanzania; August 2007—September 2008	628	Urban referral medical center and Regional hospital.	>13 years. Primarily adults	Histoplasma urine antigen testing	1 (4.0%)	7 (1.1%) positive for histoplasmosis		*Histoplasma* spp
Crump, J. A., et al[4]	Tanzania; September 2007—August 2008	870	Urban referral hospital.	Children (>2 years <13 years) Adults >13 years	Blood culture, thick and thin blood smears. Cryptococcal, *S*.*pneumoniae*, *H*.*capsulatum* antigen testing. Leptospirosis/ Brucellosis standard microscopic reciprocal test (MAT). Acute and convalescent serological investigation for Q fever and Spotted and typhus fever group rickettsiosis. NAAT for DENG, CHIKV and flavivirus RNA	11 (44.0%)	Q fever (n = 24; 5.0%) spotted fever rickettsiosis (n = 36; 8.0%) typhus group rickettsiosis (n = 2; 0.4%) chikungunya (n = 55; 7.9%) brucellosis (n = 16; 3.5%) leptospirosis (n = 40; 8.8%)		Chikungunya virus, *Leptospira*, *Rickettsial* spp,
Crump, J. A., et al[49]	Tanzania; September 2007—August 2008	403	Urban referral hospital.	>13 years. Primarily adults	Blood culture (mycobacteria), Thick and thin blood smears	2 (8.0%)	104 (25.8%) positive blood culture. 8 (2.0%) with malaria slide positive	161(39%)	*S enterica* serotype Typhi, *S*.*pneumoniae*, *E*.*coli*, *Mycobacterium tuberculosis*
Crump, J. A., et al[49]	Tanzania; September 2007—August 2008	467	Urban referral hospital.	>2 years <13 years	Blood culture. Thick and thin blood smears	2 (8.0%)	20 (4.3%) positive blood culture. 6 (1.3%) malaria slide positive	57 (12.2%)	*S enterica* serotype Typhi, *S*.*pneumoniae*, *E*.*coli*, *Plasmodium* spp
**Western Africa**									
Akpede, G. O., et al[55]	Benin; October 1988—October 1989	642	Urban referral hospital. Children's emergency room	1 month-5 years	Blood culture. Thick and thin blood smears	2 (8.0%)	24 (3.7%) positive blood culture. 403 (62.8%) malaria slide positive		*Plasmodium* spp, *S*.*aureus*
Akpede, G. O., et al[60]	Benin; October 1988—October 1989	156	Urban referral hospital. Children's emergency room	1 month-5 years	Blood culture. Thick and thin blood smears	2 (8.0%)	67 (42.9%) positive blood culture. 116 (74.4%) malaria slide positive		*Plasmodium* spp, *S*.*aureus*, *Citrobacter* spp
Ayoola, O. O., et al[61]	Nigeria; June 1998—November 1998	102	Urban referral hospital. Children's emergency room	1–12months	Blood culture. Thick and thin blood smears	2 (8.0%)	39 (38.2%) positive blood culture. 31 (30.4%) with malaria slide positive		*Plasmodium* spp, *S*.*aureus*, *E*.*coli*
Baba, M., et al[45]	Nigeria, July 2008- December 2008	310	Urban, referral, tertiary, teaching hospital	All age groups. Primarily adults	Thick and thin blood smears, Widal test. Plaque reduction neutralization tests for CHIK, YF, DENG, WNV (Did not meet case definitions for Widal and viral tests)	1 (4.0%)	49 (15.8%) malaria slide positive		*Plasmodium* spp
Ki-Zerbo, G. A., et al[57]	Burkina Faso; January 1995—March 1995	183	Teaching hospital	>15 years	Acute and convalescent serological investigation for Spotted and typhus group rickettsiosis and Q fever	2 (8.0%)	17 (5.5%)		*Rickettsial* spp (SFG) *Rickettsial* spp (TG) *Coxiella* spp
Lekweiry, K. M., et al[33]	Mauritania; 2009–2010	301	National hospital	1 month -14 years	Thick and thin blood smears, NAAT for malaria	1 (4.0%)	105 malaria positive by NAAT		*Plasmodium* spp
Obaro, S., et al[38]	Nigeria; September 2008—November 2009	969	7 hospitals	2 months -5 years	Blood culture	1 (4.0%)	111 (11.5%) with positive blood cultures		*S enterica* serotype Typhi, Non typhoidal *Salmonella*, *S*. *aureus*
**North Africa**									
Afifi, S., et al[26]	Egypt; 1999–2003	10,130	Public infectious disease hospital	> 4 years. Primarily adults	Blood culture	1 (4.0%)	1005 (10.2%) with positive blood culture		*Salmonella enterica* serotype Typhi, *Brucella* spp, *S*.*aureus*
Hyams, K. C., et al[62]	Sudan; Jan 1984—Feb 1984	100	Urban hospital	> 12 years. Primarily adults	Blood culture, virology test- isolation and acute and convalescent serological investigation for DENV, YF, WNV, CHIK, thick and thing blood smears	5 (5.0%)	25 (25%) positive blood culture, 21(21%) virus isolation, 13 (13%) malaria slide positive		Dengue virus, *Salmonella enterica* serotype Typhi, *Plasmodium* spp
**South Central Asia**									
Abbasi et al[25]	Pakistan; September 2007—January 2008	112	Urban teaching hospital	> 13 years. Primarily adults	Thick and thin blood smears. Dengue viral specific immunoglobulin detection (Did not meet dengue case definition)	1 (4.0%)	26 (23.2%) malaria slide positive		*Plasmodium* spp
Akram, D. S[63]	Pakistan; June 1994—September 1994	25	Urban, Pediatric hospital	1 month- 12 years	Acute and convalescent serology for dengue virus, West Nile virus, JEV	3 (12.0%)	10 (4%) serologically confirmed cases		Dengue virus, West Nile virus
Blacksell, S. D., et al[46]	Nepal, Kathmandu; July 2002—June 2004	103	Urban, referral, community general hospital	> 17 years	Blood culture. Serology for scrub typhus, murine typhus, leptospirosis, dengue. Included only for blood culture and paired acute and convalescent sera	3 (12.0%)	29 (28.1%) positive blood culture, 14 (13.5%) confirmed serology		*Salmonella enterica* serotype Typhi, *Salmonella enterica* Paratyphi A, *R*.*typhi*
Chrispal, A., et al[29]	South India; January 2007—January 2008	398	Tertiary care referral hospital	>16 years	Blood culture, thick and thin blood smears, serological testing for scrub typhus, Dengue virus, *Leptospira* spp, SFG rickettsiosis (did not meet serological case definitions)	1 (4.0%)	32 (8.0%) positive blood cultures, 68 malaria slide positive		Salmonella enterica serotype Typhi, *Salmonella enterica* Paratyphi A, *Plasmodium* spp
Faruque, L. I[30]	Bangladesh; December 2008—November 2009	462	Six tertiary level, teaching, referral hospital	Unspecified. Primarily adults	Malaria rapid diagnostic test. Serological testing for dengue virus (Did not meet dengue case definition)	1 (4.0%)	3 (0.6%) positive for malaria rapid diagnostic test		*Plasmodium* spp
Kaushik, J. S., et al[32]	India; June 2008—December2008	1,680	Urban tertiary, hospital	1 month- 12 years	Thick and think blood films for malaria parasites	1 (4.0%)	38 (2.3%) malaria slide positive		*Plasmodium* spp
Murdoch, D. R., et al[36]	Nepal, Kathmandu; Jan 2001—March 2001 and July—August 2001	876	Urban, general hospital	>14 years old.	Blood culture, Urinary antigen testing, serological testing for IgM antibodies dengue virus, *Leptospira* spp, Scrub typhus and *R*.*typhi* (did not meet serological case definition)	1 (4.0%)	137 (15.6%) positive blood culture		*Salmonella enterica* serotype Typhi, *Salmonella enterica* Paratyphi A
Pattanaik, Sarit S[39]	India; 2008–2009	67	Teaching hospital	>15 years.	Blood culture, NAAT	1 (4.0%)	No positive results		
Zimmerman, M. D., et al[43]	Nepal, Kathmandu; Jan 2001—March 2001 and July—August 2001	756	Urban, tertiary care hospital	>14 years old	R.typhi NAAT	1 (4.0%)	50 (6.6%) positive NAAT		*R*.*typhi*
**South East Asia**									
Archibald, L. K., et al[27]	Thailand, Bangkok; February 1997—April 1997	246	Urban, referral, infectious disease hospital.	>15 years.	Blood culture (mycobacterial)	1 (4.0%)	119 (48.4%) positive blood culture		*C*. *neoformans*, *Mycobacterium tuberculosis*, Non-typhoidal *Salmonella*
Blair, P. J., et al[28]	Cambodia; December 2006—December 2008	4,233	Two referral hospitals	> 2 years	Blood, throat and nasal specimen. rRT- NAAT, virus isolation, HI assay	1 (4.0%)	1151 (27.2%) with confirmed influenza		
Chheng, K., et al[3]	Cambodia; October 2009—October 2010	1,193	Urban, referral, government hospital.	< 16 years, neonates excluded	Blood culture. Thick and thin blood smear. Nucleic amplification test, serological testing for JEV, DENV), Acute and convalescent serological testing for *R*.*typh*i and *Orientia tsutsugamushi*, NAAT for *Leptospira* spp, nasal and throat specimen, rRT-NAAT for influenza (Did not meet case definitions for DENV and JEV)	6 (24.0%)	149 (12.5%) positive blood culture, 96 (8.0%) *Orientia tsutsugamushi*, 27 (2.2%) *R*.*typhi*, *Influenza* 25 (2.1%) 24(2.0%) malaria slide positive, 17 (1.4%) *Leptospira* spp		*Orienta tsutsugamushi*, *S*.*aureus R*.*typhi*
McDonald, L. C., et al[35]	Thailand; February 1997—March 1997 and August—September 1997	216	Urban, referral hospital in Thailand.	> 18 years	Mycobacterial blood culture	1 (4.0%)	20 (9.3%) positive blood culture	154 (71.3%) in Thailand	*Mycobacterium tuberculosis*
Cohen, Adam L[58]	Thailand; February 2002—February 2003	704	Four district rural hospitals	> 6 years. Primarily adults	Acute and convalescent serological examination for dengue virus, and *Leptospira* spp	2 (8.0%)	199 (28.3%) with confirmed serology		Dengue virus, *Leptospira* spp
Kalayanarooj, S., et al[31]	Thailand; April 1994—December 1994	172	One urban children's hospital. One rural provincial hospital	6 months—14 years	Dengue virus isolation and acute and convalescent serological examination	1 (4.0%)	60 (34.9%) with confirmed serology		Dengue virus
Wijedoru, L.P., et al[42]	Cambodia; April 2009—June 2009	134	Children's hospital	> 1 year <16 years	Blood culture	1 (4.0%)	5 (3.7%) positive blood culture		*Salmonella enterica* serotype Typhi
Libraty, D. H., et al[59]	Thailand; 1994–1999	812	One urban children's hospital. One rural provincial hospital	6 months–14 years	Acute and convalescent serological examination for *Leptospira* spp and dengue.	2 (8.0%)	468 (44.8%) with confirmed serology		Dengue virus, *Leptospira* spp
**Western Asia**									
Nimri, L. F., et al[37]	Jordan; 1998–1999	210	Urban pediatric teaching hospital.	1 month—10 years	Blood culture	1 (4.0%)	94 (44.8%) positive blood culture		*S*.*pneumoniae*, *E*.*coli*, *Klebsiella* spp

*NAAT–Nucleic acid amplification test

*spp.—species

### Bacterial and fungal bloodstream infections

Of the 45 eligible studies and 54,578 patients included in this review, blood cultures and antigen testing was conducted in 28 (62.2%) studies among 49,143 (90.0%) patients. All studies described the microbiological techniques used for blood cultures. However, the media used and methods of identification of organisms varied between studies. Minimum acceptable blood culture volumes were reported by 18 (64.3%) of 28 studies using blood cultures and ranged from 1 mL to 3mL in pediatric studies and from 5 mL to 10mL in adult studies. Of 23 studies reporting results of antimicrobial susceptibility testing, all used disc diffusion, or Epsilometer test (E-test) methods [2,3,5,26,27,35–38,46–54]. Organisms thought to be contaminants were reported as being excluded from analysis in 16 (57.1%) of the 28 studies. In six studies providing data from all positive blood cultures, contaminants were isolated from 36 (3.9%) of 920 adult blood cultures [2,50] and 107 (4.2%) of 2,550 pediatric blood cultures [3,49,51,55].

Of patients evaluated with blood culture or antigen testing, 4,852 (10.6%) were reported to have positive result. Table 4 provides a summary of the most common bloodstream isolates according to region and age group in eligible studies.

**Table 4:** Summary of eligible diagnostic tests and confirmed cases found according to region and age in all eligible studies, 1980–2013.

Disease	Eastern Africa (n = 29,286)				North Africa (n = 10,230)				Western Africa (n = 2,663)				South Central Asia (n = 4,479)				South East Asia (n = 7,710)				Western Asia				Paediatric (All Regions n = 30,295)				All Regions (n = 54,578)			
	Tested		Positive		Tested		Positive		Tested		Positive		Tested		Positive		Tested		Positive		Tested		Positive		Tested		Positive		Tested		Positive	
	N	(%) region	N	(%) tested	N	(%) region	N	(%) tested	N	(%) region	N	(%) tested	N	(%) region	N	(%) tested	N	(%) region	N	(%) tested	N	(%) region	N	(%) tested	N	(%) region	N	(%) tested	N	(%) region	N	(%) tested
Bacteria and fungal invasive infections (blood culture)	28,752	(98.1)	2,988	(10.4)	10,230	(100.0)	1,030	(10.1)	1,869	(70.2)	241	(12.8)	1,046	(23.4)	166	(15.9)	1,573	(20.4)	247	(15.7)	210	(100.0)	94	(44.8)	27,001	(89.1)	2,282	(8.5)	43,680	(80.0)	4,766	(10.9)
Gram positive			748	(2.6)			81	(0.8)			101	(5.4)			9	(0.9)			61	(3.9)			26	(12.3)			693	(2.6)			1,026	(1.9)
Streptococcus pneumoniae *			588	(2.0)			4	(>0.1)			8	(0.4)			2	(1.2)			20	(1.3)							433	(1.6)			622	(1.4)
*Staphylococcus aureus*			160	(0.6)			77	(0.8)			93	(4.9)			7	(0.7)			41	(2.6)			5	(2.4)			260	(0.9)			383	(0.9)
Gram negative			1,457	(5.0)			788	(7.7)			90	(4.8)			151	(14.4)			87	(5.5)			35	(16.7)			1,091	(4.0)			2,608	(6.0)
*Salmonella enterica*			926	(3.2)			513	(5.0)			31	(1.7)			140	(13.4)			44	(2.8)							647	(2.4)			1,654	(3.8)
Non-typhoidal *Salmonella* †			810	(2.8)			0	-			8	(0.4)							17	(1.1)							588	(2.1)			835	(1.9)
*S*. *enterica serotype* Typhimurium			267	(0.9)																											267	(0.6)
*S*. *enterica serotype* Enteritidis			121	(0.4)																											121	(0.3)
Typhoidal *Salmonella*			67	(0.3)			513	(5.0)			22	(1.2)			140	(13.4)			27	(1.7)							59	(0.2)			773	(1.8)
*S*. *enterica serotype* Typhi			63	(0.2)			508	(5.0)			22	(1.2)			75	(7.2)			27	(1.7)			4	(1.9)			59	(0.2)			614	(1.4)
*S*. *enterica serotype* Paratyphi A			4	(>0.1)			5	(>0.1)							65	(6.2)											0	-			74	(0.2)
Non-*Salmonella* Enterobacteriaceae																																
*Escherichia coli*			243	(0.8)							19	(1.0)			9	(0.9)			3	(0.2)			9	(4.3)			200	(0.7)			283	(0.6)
*Klebsiella spp*			39	(0.1)							11	(0.6)							6	(0.4)			8	(3.8)			32	(0.1)			64	(0.1)
*Enterobacter spp*			16	(0.1)							2	(0.1)			1	(0.1)							2	(1.0)			16	(0.1)			20	(>0.1)
*Citrobacter spp*			5	(>0.1)							2	(0.1)															4	(>0.1)			7	(>0.1)
*Proteus mirabilis*			4	(>0.1)							4	(0.2)															1	(>0.1)			8	(>0.1)
*Shigella spp*			6	(>0.1)																			1	(0.5)			1	(>0.1)			7	(>0.1)
Other Gram negative																																
*Brucella spp* ^¶^			1	(>0.1)			275	(2.7)																			0	-			276	(0.6)
*Haemophilus influenzae*			114	(0.4)							2	(0.1)							9	(0.6)			6	(2.9)			136	(0.5)			131	(0.3)
*Neisseria meningitidis*			36	(1.3)											1	(0.1)			4	(0.3)			4	(1.9)			23	(0.1)			45	(0.1)
*Acinetobacter* spp			17	(0.6)							14	(0.7)							3	(0.2)							17	(0.1)			34	(0.1)
*Pseudomanas* spp			19	(0.7)							4	(0.2)							4	(0.3)			1	(0.5)			14	(0.1)			28	(0.1)
*Burkholderia* spp			1	(>0.1)							1	(0.1)							14	(0.9)							0	-			16	(>0.1)
Yeasts ‡			50	(0.2)			0	-											11	(0.7)							5	(>0.1)				
*Cryptococcus* spp			43	(0.1)															10	(0.6)							3	(>0.1)			53	(0.1)
*Histoplasma* spp			7	(>0.1)															1	(0.1)							2	(>0.1)			8	(>0.1)
Other			783	(2.7)			161	(15.6)			50	(2.7)			6	(0.6)			99	(6.3)			33	(15.7)			493	(1.8)			1,132	(2.6)
Mycobacteria	1,815	(6.2)	129	(7.1)	0	-			0	-			876	(19.6)	0	-	462	(6.0)	71	(15.4)					229	(0.8)	0	-	3,153	(5.8)	200	(6.3)
*Mycobacterium tuberculosis* complex			127	(7.0)															47	(10.2)							0	-			174	(5.5)
*Mycobacterium avium* complex			2	(0.1)															24	(5.2)							0	-			26	(0.8)
Bacteria and fungal invasive infections (antigen testing)																					0	-										
Streptococcus pneumoniae *	403	(1.4)	17	(4.2)	0	-			0	-			876	(19.6)	51	(5.8)	0	-							0	-			1,279	(2.3)	68	(5.3)
*Cryptococcus* spp ‡	403	(1.4)	11	(2.7)	0	-			0	-			0	-			0	-							0	-			403	(0.7)	11	(2.7)
*Histoplasma* spp ‡	628	(2.1)	7	(1.1)	0	-			0	-			0	-			0	-							0	-			628	(1.1)	7	(1.1)
Bacterial zoonoses																																
Borrelliosis	102	(0.4)	2	(2.0)	0	-			0	-			0	-			0				0	-			0	-			102	(0.2)	2	(2.0)
Brucellosis ^¶^	453	(1.5)	16		0	-			0	-			0	-			0				0	-			246	(0.8)	5	(2.0)	453	(0.8)	16	(3.5)
Leptospirosis	453	(1.5)	40	(8.8)	0	-			0	-							2,339	(30.3)	98	(4.2)	0	-			1,881	(6.2)	50	(2.7)	2,792	(5.1)	138	(4.9)
Rickettsial infections**	552	(1.9)	41	(7.4)	0	-			183	(6.9)	9	(4.9)	756	(16.9)	50	(6.6)	1,193	(15.5)	38	(3.2)	0	-			1,679	(5.5)	56	(3.3)	2,684	(4.9)	138	(4.9)
Spotted fever group	450	(1.5)	36	(8.0)					183	(6.9)	7	(3.8)	0	-											243	(0.8)	18	(7.4)	633	(1.2)	43	(6.8)
Typhus group	450	(1.5)	2	(0.4)					183	(6.9)	2	(1.1)	756	(16.9)	50	(6.6)	1,193	(15.5)	27	(2.3)					1,436	(4.7)	27	(1.9)	2,582	(4.7)	89	(3.3)
Unspecified *Rickettsia* spp	102	(0.4)	3	(3.0)					0				0	-					11	(0.9)					0	-	11*	(0.9)	102	(0.2)	14	(13.7)
Scrub typhus	0	-			0	-			0	-			103	(2.3)	5	(4.9)	1,193	(15.5)	96	(8.0)	0	-			1,193	(3.9)	96	(8.0)	1,296	(2.4)	101	(7.8)
Q fever	482	(1.6)	24	(5.0)	0	-			183	(9.8)	8	(4.4)	0	-			0				0	-			268	(0.9)	7	(2.6)	586	(1.1)	32	(5.4)
Blood parasites																																
Malaria	6,789	(23.2)	2,659	(39.1)	100	(1.0)	13	(13.0)	1,511	(56.7)	657	(43.5)	2,652	(59.2)	135	(5.1)	1,193	(15.5)	24	(2.0)	0	-			9,030	(29.8)	926	(10.3)	12,245	(22.4)	3,488	(28.5)
Viral infections																																
Influenza	0	-			0	-			0	-			0	-			5,426	(70.4)	1,176	(21.7)	0	-			1,193	(3.9)	25	(2.1)	5,426	(9.9)	1,176	(21.7)
Dengue	700	(2.4)	0	-	100	(1.0)	21	(21.0)	0	-			25	(0.6)	9	(36.0)	1,688	(21.9)	542	(32.1)	0	-			1,341	(4.4)	419	(31.2)	2,513	(4.6)	572	(22.8)
West Nile	700	(2.4)	0	-	100	(1.0)	0	-	0	-			25	(0.6)	1	(4.0)	0	-			0	-			332	(1.1)	0	-	825	(1.5)	1	(0.1)
Chikungunya	700	(2.4)	55	(7.9)	100	(1.0)	0	-	0	-			0	-			0	-			0	-			332	(1.1)	34	(10.2)	800	(1.5)	55	(6.9)
Yellow fever	700	(2.4)	0	-	100	(1.0)	0	-	0	-			0	-			0	-			0	-			332	(1.1)	0	-	800	(1.5)	0	-
Japanese encephalitis	0	-			0	-			0	-			25	(0.6)	0	-	0	-			0	-			0	-			25	(>0.1)	0	-

* *Streptococcus pneumoniae* was tested by both blood culture and urine antigen testing, table number of patients tested using each method from each geographical region and positive results from each.

† Non-typhoidal *Salmonella* and Typhoidal *Salmonella* were not consistently described to species level, thus total reported number for each group was greater than sum of species. *Brucella* spp was tested by both blood culture and serological methods, table number of patients tested using each method from each geographical region and positive results from each.

‡ Yeasts (*Cryptococcus* spp, *Histoplasma* spp) were tested by both blood culture and antigen testing, table number of patients tested using each method from each geographical region and positive results from each.

** *Rickettsia* spp were not consistently described to species level, one study from South East Asia testing for typhus group rickettsiosis reported 11 unspecified *Rickettsia* spp. were identifie

Antimicrobial use before admission was assessed in 14 studies and 13,805 patients, and ranged from 9 (8.7%) of 103 to 111 (47.6%) of 233 [6,26,27,35,36,38,42,46–50,52,56]. Of nine studies evaluating the role of pre-admission antimicrobial exposure on blood culture positivity, five (55.5%) showed that pre-enrolment use of antimicrobials was not associated with a blood culture being positive [36,47,48,50,56]. One (11.1%) study showed a statistically significant increase in blood culture positivity [27], and two (22.2%) study identified fewer positive blood cultures in patients previously treated with antimicrobial drugs than in those who were not [42,49].

### Bacterial zoonoses

Out of the 45 eligible studies with 54,578 patients, bacterial zoonoses were investigated in nine (20.0%) and included 14,773 (27.6%) patients. Of the 18,142 tests for seven bacterial zoonotic diseases (borreliosis, brucellosis, leptospirosis, spotted fever and typhus group rickettsiosis, scrub typhus, and Q fever) 702 (3.9% of tests) met the case definition (Table 4).

Of nine studies investigating bacterial zoonoses, six (66.7%) studies met our pre-defined testing criteria for rickettsial infection [3,4,43,44,46,57] and five (55.5%) studies met the pre-defined criteria for leptospirosis [3,4,44,58,59]. In one study from South East Asia investigating febrile children at a tertiary referral urban children’s hospital and a rural provincial hospital, of 14 confirmed leptospirosis cases, 10 (71.4%) were from patients at the rural hospital [59].

### Malaria and other blood parasites

Of all studies included in this review, 24 (51.1%) reported the prevalence of malaria parasites identified by thick or thin smear, nucleic acid amplification test (NAAT), or rapid diagnostic test. No study reported the detection of blood parasites other than malaria.

Of all 54,578 patients in this review, 12,245 (22.4%) were enrolled in the 23 studies testing for malaria, of which 3,488 (28.5%) had a positive result according to our laboratory-based case definitions (Table 4) [2,3,25,29,30,32,33,44,45,47–56,60–62].

Of studies testing for malaria, 13 (54.2%) were conducted in Eastern Africa. Of the 12,245 patients tested for malaria, 10,535 (86.0%) were tested using thick or thin malaria blood smears only and one study tested 462 (3.8%) patients for malaria using rapid diagnostic tests only [30]. Three studies used two or more tests to diagnose malaria [41,44,53]. Among the 3,106 *Plasmodium* spp. that were identified to the species level, 2,928 (94.3%) were *Plasmodium falciparum*, of which 2,907 (99.3%) were found in the African regions. *Plasmodium vivax* accounted for 173 (5.6%) of speciated *Plasmodium* spp. Of the 92 *Plasmodium* spp. identified among patients in South Central and South East Asia 75 (81.5%) were *Plasmodium vivax*.

### Viral infections

Of the 45 eligible studies with 54,578 patients in this review, viral infections were investigated according to our laboratory case definitions in eight (17.0%) studies including 7,939 (14.4%) patients using 10,389 tests for the six infections; chikungunya, dengue, influenza, Japanese encephalitis, West Nile virus infection, and yellow fever virus (Table 4).

Of viral infections, dengue fever was the most commonly assessed and was investigated using an eligible test in six (12.8%) studies; one (20.0%) each in Eastern Africa [4], North Africa [62], South Central Asia [63] and three (50.0%) studies in South East Asia [3]. In total 2,513 (4.6%) patients were tested for dengue fever using virus isolation, NAAT, or serology according to our case definitions.

### HIV co-infection

Of the 15 studies with 9,365 patients that included HIV testing, 1,988 (21.2%) patients were found to be HIV seropositive. There were insufficient data in included studies to investigate the association between HIV and infections other than bacterial and fungal bloodstream infection. In nine studies with adequate data for analysis [2,6,27,35,47,49,52,64],1,667 (59.4%) of 2,805 patients with HIV infection had bloodstream infections versus 1,357 (52.8%) of 2,566 HIV-uninfected patients (OR 1.3, 95% CI = 1.2–1.5, p<0.0001) (Table 5).

**Table 5:** Causes of bloodstream infection by HIV serostatus in nine fever etiology studies in low- and middle-income countries, 1980–2013.

Blood culture isolate	Total isolates (% with BSI)		Patients infected with HIV (% with BSI)		Patients not infected with HIV (% with BSI)		OR for those infected with HIV	
	N	(%)	N	(%)	N	(%)	OR	P-value
*Mycobacterium* spp.	241	(39.0)	237	(38.3)	4	(0.6)	44.5	p<0.0001
*Streptococcus pneumoniae*	82	(15.1)	56	(10.3)	26	(4.8)	2.9	p<0.0001
Non-typhoidal *Salmonella*	60	(13.8)	54	(12.4)	6	(1.4)	16.5	p<0.0001
*Salmonella enterica* Typhi	34	(10.8)	2	(0.6)	32	(10.1)	0.12	p<0.05
*Escherichia coli*	29	(6.3)	14	(3.0)	15	(3.2)		NS
*Staphylococcus aureus*	25	(5.6)	13	(2.9)	12	(2.7)		NS
**Total bloodstream infection**	3,024	-	1,667	**(55.1)**	1,357	(44.9)	1.3	**p<0.001**

### Clinical diagnosis

Five studies, four from Eastern Africa [4,44,47,51,53,56] and one from South Central Asia [46], provided sufficient data regarding both clinical and laboratory confirmed diagnoses for the cause of fever and enabled assessment the accuracy of clinical diagnosis. In Eastern Africa a clinical diagnosis of malaria was recorded in 800 (51.1%) of 1,566 patients of whom 85 (5.4%) had malaria parasites identified through laboratory diagnostic testing. In the study from South Central Asia 52 (50.5%) of 103 patients presenting with fever had a clinical diagnosis of enteric fever. Of the 52 patients with a clinical diagnosis of enteric fever 20 (38.5%) were found to have a positive blood culture for typhoidal *Salmonella* [46].

### Concurrent infections

Seven (15.6%) out of the 45 eligible studies provided information regarding apparent concurrent infections. Of 5,719 patients enrolled in studies reporting such information, 198 (3.5%) were found to have both a positive blood culture for a pathogen and a positive malaria smear [2,48,52,53,55,60,61].

Moreover, one study in South Central Asia showed evidence for mixed infections of *S*. *enterica* serotype Typhi with scrub typhus or typhus group rickettsiosis [46].

### In-hospital case fatality ratio

Of all 45 studies, 16 (35.6%) including 10,756 patients provided sufficient data regarding in-hospital case fatality ratio [4–6,26,27,32,36,40,41,47,48,53,55,56,61,62]. Of 10,756 patients, 1,307 (12.2%) patients died during their hospital stay. Cause of death information was provided in 12 studies [5,6,26,27,32,40,41,47,48,53,55,56,61].

### Seasonal variation

Associations between seasons and the prevalence of febrile illnesses was reported in 11 studies [26,28,30,33,36,40,43,47,55,59]. Of the 47 eligible studies, 18 (38.3%) were conducted for one year or longer. The median (range) study duration was 6 (1 to 60) months.

Data from two Malawian cohorts showed a shift from a predominance of non-typhoidal *Salmonella* in blood cultures during the wet season to a predominance of *S*. *pneumoniae* during the dry season [5,47]. A Mozambique study found no association between season and bloodstream infections [40].

A study from Egypt suggested an association between the onset of the rainy season and a predominance of *S*. *enterica* serotype Typhi isolates. In the same study it was shown that brucellosis was reported in all months of the year with peaks in the spring and early summer, coinciding with the parturient seasons of domestic animals [26].

In Nepal *S*. *enterica* serotype Typhi and Paratyphi A were the most common bloodstream isolates during both monsoon and winter seasons. However, there was a substantial increase in the proportion of *Salmonella* Paratyphi A isolates during the monsoon season. In another study done in Nepal, murine typhus was more common during the winter season than the summer [43].

Vector-borne and zoonotic diseases such as dengue and leptospirosis were found to be more common during the rainy seasons in Mauritania and Thailand [33,59]. In Cambodia, influenza virus cases peaked during the rainy season [28].

## Discussion

To our knowledge, this is the first systematic review of severe febrile illness etiology for a broad range of pathogens in all LMICs. We show that bacterial and fungal bloodstream infections, bacterial zoonoses, malaria, and viral infections are leading causes of severe febrile illness, and that their relative importance appears to vary by geographic region (Table 4). Our findings confirm that some infectious causes of fever are closely linked to HIV co-infection, that severe febrile illness is associated with high in-hospital case fatality ratios, that some pathogens show seasonal patterns, and that clinical diagnosis is unreliable among febrile patients, especially for pathogens causing systemic infections. Most notably, we demonstrate that there are major gaps in our current understanding of the causes of severe febrile illness in LMICs. Some potentially important pathogens have not been rigorously studied in any country, many studies examined only one or a few pathogens, many countries and some geographic regions had no eligible research on severe febrile illness etiology, and representation of age groups was inconsistent.

Bacterial and fungal bloodstream infections were the most sought febrile disease with a total of 30 (63.8%) out of 47 studies included in this review conducting blood cultures, 22 (47%) of which were conducted in the African continent. Overall, the proportion of severe febrile illness attributed to invasive bacterial and fungal infections was 10.7%, 8.5% among children and 13.9% among adults. Although our ability to examine geographic and age-related patterns of bloodstream infections was limited by incomplete representativeness of studies, some observations are possible. *Salmonella enterica* was the most common bloodstream isolate. Non-typhoidal *Salmonella* (NTS) predominated in all African regions, except for Northern Africa where *Salmonella* Typhi was more common. In Asian regions *Salmonella* Typhi and *Salmonella* Paratyphi A predominated. *S*. *pneumoniae* was the most common Gram-positive invasive infection in both African and Asian regions. *S*. *pneumoniae* was particularly common in paediatric cohorts in both areas accounting for 19.2% of bacteraemia in the African regions and 16.5% in the Asian regions. Fastidious organisms, such as *S*. *pneumoniae*, may be less often isolated than those without special growth requirements. This may have affected the relative prevalence of different species in our review.

*Plasmodium* spp. was the most commonly identified organism among patients with febrile illness overall. As expected, *Plasmodium falciparum* predominated in the African regions while *Plasmodium vivax* predominated in Asian regions. Malaria parasite and bacterial bloodstream co-infections were common among patients with positive malaria diagnostic tests especially in areas with year-round intense malaria transmission [2,40,48,51,53,55,60,61]. It is apparent that *Plasmodium* spp. may act as the prime pathogen; as a co-pathogen, increasing risk for other infections such as NTS bacteremia in some circumstances [65]; or as an bystander in others. In the latter, despite having a positive blood film the patient is suffering from another illness and the parasitemia, that should nonetheless also be treated, is incidental [66].

Studies that used an afebrile control group to calculate the fraction of febrile illness attributable to malaria indicate that incidental parasitemia is likely to be particularly common in malaria-endemic areas [2,47,64]. Such studies could be improved by including routine measurement of malaria parasite density. Furthermore, studies comparing clinical diagnosis with laboratory diagnosis of malaria confirmed that malaria over-diagnosis is common among patients with severe febrile illness. Incidental *Plasmodium* spp. infection and malaria over-diagnosis increase the risk of the patient not being treated for the actual cause of the current illness [4,8,44,47,51,56,64].

With respect to bacterial zoonoses and viral infections, large geographical areas had no or few studies, and few patients were evaluated for these infections. Those studies that did evaluate for bacterial zoonoses and viral infections varied widely in pathogens sought and diagnostic tests used. Many studies did not collect convalescent serum, precluding conventional standard diagnostic testing and therefore inclusion in our review. Among eligible studies, case fractions were found to be highly variable across regions and age groups and the small number of both studies and participants suggest that prevalence data should be interpreted with caution. Among eligible studies, spotted fever rickettsiosis predominated in African regions, with brucellosis being common in Northern Africa, while typhus group rickettsiosis, scrub typhus, and leptospirosis were particularly common in Asia. Among viral infections, dengue fever was found to be an important cause of febrile illness in Asia and was associated with a high case ratio.

No eligible studies were found from Latin America and the Caribbean, Oceania, some regions of Africa. There were a small number of studies from populous regions of Asia. Furthermore, the majority of LMICs did not have a single eligible study. In addition, we found that there was a limited amount of research in rural settings, despite the majority of countries searched in this review having predominantly rural populations [67]. Future research studies should improve geographic representativeness and include rural study sites.

This systemic review had a number of limitations. We included only studies of severe febrile illness that required admission to hospital emergency or inpatient departments. It is likely that patterns of infection could be quite different in outpatient, primary care, and community settings [68]. We included studies from 1980, just prior to the onset of the global HIV pandemic. We may have missed potentially relevant studies done in earlier years. It is also possible that patterns of infection in the 1980s and 1990s do not reflect the contemporary picture. Because we used conventional standard laboratory-based case definitions, some infections causing the most severe illness resulting in death before acquisition of a convalescent serum sample could not be ascertained. We did not collect data systematically on localised infections among febrile patients. However, such data was rarely reported in the studied included in this review.

Many studies did not enroll all age groups. This meant that age-related differences in severe febrile illness etiology could not be assessed. HIV infection appears to increase risk for a number of infections that may present with severe febrile illness, such as cryptococcal disease, bacteremic disseminated tuberculosis, and NTS bacteremia [2,6,27,35,47,49,52,64]. However, there were insufficient data to assess the role of HIV co-infection for a number of other pathogens evaluated in this study. Several studies found seasonal patterns with some pathogens. However, not all studies ran for a full year and others that did include at least a year of enrollment did not explore seasonality. Ideally, fever etiology research should include all age groups, at least one annual cycle, and routinely assess HIV infection status of participants. Furthermore, while we were restrictive with laboratory case definitions, we were unable to account for variability in some aspects of clinical and laboratory practice at study sites. Finally, data were aggregated by region by combining individual patient results across studies, resulting in a greater influence of larger studies.

We suggest that the current understanding of the etiology of severe febrile illness in LMICs is incomplete. High quality severe febrile illness etiology research that is comprehensive with respect to pathogens and geographically representative could improve patient outcomes by informing patient management guidelines and disease control priorities [69,70]. We recommend that multi-center severe febrile illness research should investigate a broad range of treatable or preventable infections; use standardized and quality assured diagnostic tests with rigorous case definitions; include healthy community controls to allow accurate estimations of attributable case fractions [71,72]; be geographically and demographically representative; have standardized reporting of fever associated with localized infections and should cover at least a full calendar year to incorporate seasonal variation. Such information is an essential component of an effective health system but the gaps in evidence identified by this study are likely to require coordinated resources and expertise to fill in LMICs.

## Supporting Information

S1 Fig Full-text articles reviewed but excluded. (XLS)

S2 Fig Research protocol. (DOC)

S3 Fig PRISMA checklist. (DOC)
