# Supplementary material for: Etiology of Severe Febrile Illness in Low- and Middle-Income Countries: A Systematic Review
Source: PLoS One. 2015 Jun 30;10(6):e0127962. doi: 10.1371/journal.pone.0127962 (PMC4488327; doi:10.1371/journal.pone.0127962)
Supplement: S2 Fig — (DOC) [file pone.0127962.s002.doc]

**RSEARCH PROTOCOL**

**Etiology of severe febrile illness in low- and middle-income countries: a systematic review**

**INVESTIGATORS**

Namrata Prasad, BBiomedSc, DPH^1^

David R. Murdoch, MB ChB, MD, MSc, DTM&H^2^

Hugh Reyburn, MB BS, MSc, DTM&H^3^

John A. Crump, MB ChB, MD, DTM&H^1^

^1^Centre for International Health, Dunedin School of Medicine, University of Otago, PO Box 56, Dunedin 9054, New Zealand

^2^Department of Pathology, University of Otago Christchurch, 2 Riccarton Avenue, PO Box 4345, Christchurch 8011, New Zealand

^3^Department of Disease Control, Faculty of Infectious and Tropical Diseases, London School of Hygiene and Tropical Medicine, Keppel Street, London WC1E 7HT, United Kingdom

**VERSION 1.0**

**6 April 2014**

**BACKGROUND AND RATIONALE**

Febrile illness is one of the most common reasons for presentation to hospitals in low- and middle-income countries (LMIC) worldwide and is associated with considerable morbidity and mortality . Unlike the syndromes of pneumonia and diarrhea which have well defined global burden of disease estimates, a comprehensive approach has not been taken for investigating the major infectious causes of febrile illnesses . Instead, studies have tended to focus on one pathogen or a small group of pathogens, or have concentrated on the use of a single diagnostic test.

Illness and death due to malaria is well understood , albeit subject to criticism . Increasingly data are available on the role of bacterial and fungal bloodstream infections as causes of severe febrile illness . However, fewer data are available on other causes of febrile illness including bacterial zoonoses such as leptospirosis and viral infections such as chikungunya . Furthermore, global burden of disease estimates provide envelopes of illness and death for pneumonia and diarrhoea available for allocation to individual pathogens, whereas no such envelope exists for febrile illness .

Further challenging the study of febrile illness are the non-specific symptoms and signs that accompany its many causes. In resource-limited settings where such illnesses are particularly common, health care workers often must rely on diagnosis to the syndrome level only, assumptions regarding circulating pathogens that are rarely based on local data, and empiric treatment strategies.

It is evident that a robust contemporary picture of treatable and preventable infectious causes of severe febrile illness is urgently needed to improve patient outcomes and to inform disease control efforts in LMICs. To describe epidemiologic patterns and to identify data gaps in our understanding of severe febrile illness in low resource areas, we aim to conduct a systematic review analysing prospective hospital-based studies of the etiology of febrile illness in LMICs.

**STUDY GOALS AND OBJECTIVES**

1. To provide an insight into the various etiological agents causing severe fever and to estimate the case fraction of specific infections among febrile patients admitted to hospital.

2. To identify information gaps that exist regarding diagnostic testing and evaluations of febrile diseases.

**STUDY DESIGN**

The systematic review will be done according to the Preferred Reporting Items for Systematic Reviews and Meta-Analyses (PRISMA) guidelines .

**Geographic and human development classification of countries**

Countries will be categorized into areas and regions according to the United Nations Population Division classification (Table 1) . From each region, low- and middle-income countries will be selected according to the 2012 Human Development index (HDI) .

**Pathogens, diseases, and case definitions**

A list of pathogens and diseases associated with febrile illness in low- and middle-income countries and their associated case definitions based on laboratory confirmation will be created for the search.

**Search strategy and selection criteria**

Three main databases: Ovid Medline, Scopus, and Web of Knowledge will be used for the search strategy. The search will include articles in all languages and will be limited to articles investigating humans published from the year January 1980 through to July 2013.

Search terms will be identified and defined with the assistance of an academic liaison librarian (Sarah Gallagher). With the aim of the search being to locate all prospective, hospital-based studies conducted in United Nations Development Programme (UNDP)-classified low- and middle-income countries worldwide during the time period of January 1980 through July 2013. Online translation tools will be used to evaluate non-English titles, abstracts, and full text articles.

**Title and abstract review**

One investigator will review titles and abstracts of articles identified by the search strategy. Those that appear to be prospective studies of consecutive febrile patients enrolled in the emergency department or inpatient service of hospitals in an LMIC during the time period 1980 through 2013 will be selected for full-text review.

**Full-text review**

Two investigators will reviewed full-text articles identified by the title and abstract review. A third investigator will serve as tiebreaker, independently reviewing articles to resolve disagreement between the other two investigators. To be eligible for data extraction, full-text articles were confirmed to be prospective studies of consecutive febrile patients enrolled in the emergency department or inpatient service of hospitals in a low- or middle-income country during the time period 1980 through 2013. For the purposes of this review, febrile patients were defined as a person with a history of fever in the past 48 hours; an axillary temperature ≥37·5°C; or a rectal temperature ≥38·0°C. In addition, participants in such studies needed to be evaluated for at least one of the febrile diseases of interest using laboratory-confirmed case definitions.

**Data management and statistical analysis**

Data extraction from each eligible study will be conducted by one investigator (NP) and will include: geographical location of the healthcare facility; healthcare facility rurality; study time dates and duration; study inclusion and exclusion criteria including age range; diagnostic techniques for each infection; number of patients tested for each infection; number tested meeting case definition for each infection; use of additional tests (e.g., HIV serology). When available, clinical diagnosis of patients; in-hospital fatality ratio; seasonal variation of pathogens; and pre-admission use of antimicrobials will also be recorded. For the purpose of this review, pediatric studies will be defined as those that included patients aged from ≥28 days to <15 years. Studies with mixed populations of adults and children will be analyzed as adult studies. Queries regarding data extraction will be resolved by return to the original manuscript by three investigators.

Following data extraction, infections will be organized into four groups: blood parasites; bacterial and fungal bloodstream infections; bacterial zoonoses; and viral infections, as shown in Table 2. Data from all individuals in all studies will be aggregated to compare prevalence of febrile diseases across studies and regions. Analyses of associations between patient factors or clinical conditions (e.g., HIV infection) and specific febrile diseases (e.g., cryptococcal disease) will be done for studies in which full data is available for both the pathogens and factors being assessed.

**REFERENCES**

1. Feikin DR, Olack B, Bigogo GM, Audi A, Cosmas L, et al. (2011) The burden of common infectious disease syndromes at the clinic and household level from population-based surveillance in rural and urban Kenya. PLoS One 6: e16085.

2. Farr NM, Nelson BD (2014) Child Mortality in Developing Countries. The MassGeneral Hospital for Children Handbook of Pediatric Global Health: Springer. pp. 3-12.

3. Liu L, Johnson HL, Cousens S, Perin J, Scott S, et al. (2012) Global, regional, and national causes of child mortality: an updated systematic analysis for 2010 with time trends since 2000. The Lancet 379: 2151-2161.

4. Murray CJ, Rosenfeld LC, Lim SS, Andrews KG, Foreman KJ, et al. (2012) Global malaria mortality between 1980 and 2010: a systematic analysis. The Lancet 379: 413-431.

5. Todd J, De Francisco A, O'dempsey T, Greenwood BM (1993) The limitations of verbal autopsy in a malaria-endemic region. Ann Trop Paediatr 14: 31-36.

6. Lynch M, Korenromp E, Eisele T, Newby H, Steketee R, et al. (2012) New global estimates of malaria deaths. The Lancet 380: 559.

7. Deen J, von Seidlein L, Andersen F, Elle N, White NJ, et al. (2012) Community-acquired bacterial bloodstream infections in developing countries in south and southeast Asia: a systematic review. Lancet Infect Dis 12: 480-487.

8. Reddy EA, Shaw AV, Crump JA (2010) Community-acquired bloodstream infections in Africa: a systematic review and meta-analysis. Lancet Infect Dis 10: 417-432.

9. Acestor N, Cooksey R, Newton PN, Menard D, Guerin PJ, et al. (2012) Mapping the aetiology of non-malarial febrile illness in Southeast Asia through a systematic review—terra incognita impairing treatment policies. PLoS One 7: e44269.

10. Crump JA, Morrissey AB, Nicholson WL, Massung RF, Stoddard RA, et al. (2013) Etiology of Severe Non-malaria Febrile Illness in Northern Tanzania: A Prospective Cohort Study. PLoS Negl Trop Dis 7.

11. Lozano R, Naghavi M, Foreman K, Lim S, Shibuya K, et al. (2013) Global and regional mortality from 235 causes of death for 20 age groups in 1990 and 2010: a systematic analysis for the Global Burden of Disease Study 2010. The Lancet 380: 2095-2128.

12. Moher D, Liberati A, Tetzlaff J, Altman DG, PRISMA Group (2009) Preferred reporting items for systematic reviews and meta-analyses: the PRISMA statement. Ann Intern Med 151: 264-269.

13. Liberati A, Altman DG, Tetzlaff J, Mulrow C, Gøtzsche PC, et al. (2009) The PRISMA statement for reporting systematic reviews and meta-analyses of studies that evaluate health care interventions: explanation and elaboration. Ann Intern Med 151: W65-94.

14. United Nations Population Divison Classification of Countries by major area and region of the world. In: Department of Economic and Social Affairs, editor.

15. United Nations Development Project (UNDP) (2012) Human Development Report 2013.
